# Supplementary material for: Exploring Partners’ Part in Shaping the Home Food Environment During the Transition to Fatherhood
Source: Nutrients. 2024 Dec 17;16(24):4356. doi: 10.3390/nu16244356 (PMC11679244; doi:10.3390/nu16244356)
Supplement: Supplementary file 1 [file nutrients-16-04356-s001.zip › nutrients-3332207-supplementary.pdf]

Consolidated criteria for reporting qualitative studies (COREQ): 32-item checklist.

Developed from: Tong A, Sainsbury P, Craig J. Consolidated criteria for reporting qualitative research (COREQ): a 32-item checklist for interviews and focus groups. International Journal for Quality in Health Care. 2007. Volume 19, Number 6: pp. 349 – 357

Number	Item	Guide questions/description	
**Domain 1: Research team and reflexivity**			
	Personal Characteristics		
1	Interviewer/facilitator	Which author/s conducted the interview or focus group?	CP
2	Credentials	What were the researcher's credentials?	CP: RD, MSc Population Health<br>MR: MD, DCH, FRCPCH<br>NS: MSc Nutrition, PhD Psychology
3	Occupation	What was their occupation at the time of the study?	CP : PhD student.<br>MR: Professor emeritus, Population Health<br>NS: Senior lecturer, Health Care
4	Gender	Was the researcher male or female?	Female
5	Experience and training	What experience or training did the researcher have?	CP is a Clinical dietitian with 25 years of experience, especially with women and mothers of preschool children.<br>MR is a paediatrician and professor of public health<br>NS experienced researcher and psychotherapist, hold an MSc in Nutrition. Completed training in Oxford University for Qualitative work and published work in this area.
	Relationship with participants		
6	Relationship established	Was a relationship established prior to study commencement?	Researchers had no relationship with participants prior to the commencement of the study.
7	Participant knowledge of the interviewer	What did the participants know about the researcher?	Participants knew PC was a researcher, and the organisations to which she was affiliated for the purposes of this study.
8	Interviewer characteristics	What characteristics were reported about the interviewer/facilitator?	Authors have all acknowledged their places of work and affiliations. The participants were aware that the researchers aimed to assist young families in enhancing their health.
**Domain 2: Study design**			
	Theoretical framework		
9	Methodological orientation and theory	What methodological orientation was stated to underpin the study?	Thematic Content Analysis.

	Participant selection		
10	Sampling	How were participants selected?	Purposive Sampling.
11	Method of approach	How were participants approached?	Expectants couples were recruited using advertisements posted on social media websites and printed flyers.
12	Sample size	How many participants were in the study?	15 male partners of first-time pregnant women completed an interview and questionnaire during pregnancy.<br>12 of them completed a second interview and 14 completed the second questionnaire as fathers.
13	Non-participation	How many people refused to participate or dropped out? Reasons?	3 participants who contacted us in week 38 or later were not eligible for the study.<br>3 fathers did not participate in the second interviews as 2 were ill but did complete the second questionnaire.
	Setting		
14	Setting of data collection	Where was the data collected?	Individual face to face interviews in the participants' homes + online questionnaires<br>Some of the interviews were conducted online via videoconferencing due to the covid pandemic
15	Presence of non-participants	Was anyone else present besides the participants and researchers?	No, all were individual face to face interviews.
16	Description of sample	What are the important characteristics of the sample?	Participants' mean age was 27.7 years and gestation 28 weeks. All had completed high school and were healthy, four reported BMI >25. They came from a range of backgrounds.
	Data collection		
17	Interview guide	Were questions, prompts, guides provided by the authors? Was it pilot tested?	Interview topic guide was developed and reviewed by all authors.<br>Topic guide was piloted with 5 pregnant women prior to the study.
18	Repeat interviews	Were repeat interviews carried out? If yes, how many?	12 repeated interviews were carried out.<br>They took place between 6 to 14 months postpartum.
19	Audio/visual recording	Did the research use audio or visual recording to collect the data?	Yes, audio-visual recordings.<br>+ photos of the PHFE in kitchens.
20	Field notes	Were field notes made during and/or after the interview or focus group?	Yes, field notes were made during and after each interview and after each questionnaire.
21	Duration	What was the duration of the interviews or focus group?	Interviews lasted for approximately 50 min each. Questionnaires for about 20 minutes each.
22	Data saturation	Was data saturation discussed?	Yes.
23	Transcripts returned	Were transcripts returned to participants for comment and/or correction?	No.
**Domain 3: Analysis and findings**			
	Data analysis		

24	Number of data coders	How many data coders coded the data?	PC coded the data, NS coded sub sample of data data, to discuss emerging themes and check for agreement
25	Description of the coding tree	Did authors provide a description of the coding tree?	Yes.
26	Derivation of themes	Were themes identified in advance or derived from the data?	Themes were derived from the data.
27	Software	What software, if applicable, was used to manage the data?	Excel sheets.
28	Participant checking	Did participants provide feedback on the findings?	No.
	Reporting		
29	Quotations presented	Were participant quotations presented to illustrate the themes / findings? Was each quotation identified?	Yes, Quotes are provided in an anonymized personal format, with the addition of specifying the timing of the interview, either before or after the birth.
30	Data and findings consistent	Was there consistency between the data presented and the findings?	Yes.
31	Clarity of major themes	Were major themes clearly presented in the findings?	Yes.
32	Clarity of minor themes	Is there a description of diverse cases or discussion of minor themes?	Yes.
